# Determination of the Tool–Chip Contact Length for the Cutting Processes

**DOI:** 10.3390/ma15093264

**Published:** 2022-05-02

**Authors:** Michael Storchak, Konstantin Drewle, Christian Menze, Thomas Stehle, Hans-Christian Möhring

**Affiliations:** Institute for Machine Tools, University of Stuttgart, Holzgartenstraße 17, 70174 Stuttgart, Germany; konstantin-drewle@t-online.de (K.D.); christian.menze@ifw.uni-stuttgart.de (C.M.); thomas.stehle@ifw.uni-stuttgart.de (T.S.); hans-christian.moehring@ifw.uni-stuttgart.de (H.-C.M.)

**Keywords:** cutting, contact length, FE cutting model, simulation, constitutive equation parameters

## Abstract

The thermomechanical interaction of the tool with the chip in the most loaded secondary cutting zone depends on the contact length of the tool rake face with the chip. Experimental studies of the dependency of the contact length on the cutting speed, the undeformed chip thickness, and the tool rake angle, performed by the optical method, are used for comparison with the contact length obtained by the FE modeling of the orthogonal cutting process. To determine the parameters of the constitutive Johnson–Cook equation, which serves as a material model of the FE cutting model that has a predominant influence on the contact length, a software-implemented algorithm was developed. This algorithm is based on determining the generalized parameters of the constitutive equation through finding the intersection of these parameter sets. The plurality intersection of the parameter sets of the constitutive equation is determined by means of the design of experiments and refined by subsequent multiple iterations. The comparison of the contact length values, obtained by simulating the cutting process using the generalized parameters of the constitutive equation as a material model with their experimental values, does not exceed 12% for a wide range of cutting speeds and depths of cut, as well as for the tool rake angle.

## 1. Introduction

Cutting is one of the most common processes for forming various products. The cutting process is manifested externally by different physical quantities that can characterize the process. The most important characteristics of the cutting process are the thermomechanical properties of the process, such as the forces and temperatures, the chip morphology, and the chip compression value, as well as the micro-geometry and the physical–mechanical parameters of the machined material surface. The cutting process is externally expressed by various physical quantities that can characterize the process. The most important characteristics of the cutting process are the thermomechanical properties [1,2,3], such as the forces and temperatures, the chip morphology, and the chip compression value, as well as the micro-geometry and the physical–mechanical parameters of the machined material surface. These quantities are generally used to determine the state of the cutting process. In addition to the properties of the technological system that realize the cutting process, the contact parameters between the tool, chip and workpiece also define the cutting process. An important contact characteristic is the contact length between the rake face of the tool and the chip, which determines the interaction in the secondary cutting zone, e.g., the friction and heat transfer between the tool and the chip. The contact length has a complex relationship with the parameters and conditions of the cutting process, the thermomechanical properties of the machined material, the cutting tool geometry, and others. Due to the complexity of these relationships, it is not yet possible to analytically determine the contact length between the rake face of the tool and the chip. Simultaneously, the contact length is used as a reference value in many analytical cutting models; see, e.g., [1,4,5]. Furthermore, the contact length is used as a verification value of simulation results for the numerical cutting models. It can serve as a reference point for evaluating the correct selection of parameters of the constitutive equation and the parameters of the friction model for use in the numerical cutting models. Determining the contact length between the rake face of the tool and the chip is, therefore, an important issue, and solving it will improve the accuracy and validity of the modeling results of various cutting processes. This paper deals with experimental and simulative studies to determine the contact length for further use in analytical and numerical cutting models.

## 2. Methods for the Determination of Contact Length

The cutting process takes place under complex deformation and contact interactions between the tool and the workpiece. The length of the chip–tool interaction in the direction of flow is called the natural contact length [6]. Within this contact length, there are many thermomechanical interactions between the rake face of the cutting tool and the chip [1,3]. Because of this, a heat flow occurs, which is why the natural contact length has a significant influence on the resulting tool temperature [7]. An increased temperature accelerates chemical reactions, abrasive wear, and diffusion, which in turn increase tool wear [6,8,9,10]. Therefore, the theoretical and experimental description or investigation of the natural contact length has a long historical development, and the major part of the investigations presented in the literature to determine the contact length are carried out within the framework of a fundamental understanding of the cutting process as well as the tribological aspects, such as the friction and wear between the tool and chip [11,12,13,14]. Lee and Shaffer [15] derived an equation for the determination of the natural contact length, considering the uncut chip thickness, the shear angle, and the rake angle. Abuladze [16] developed a model of the contact length dependence on the uncut chip thickness, the shear angle, and the compression ratio. Bhattacharyya [17] used natural contact length and controlled-contact cutting tools to explain the character of the influence of a small amount of lead on the metallic friction phenomenon in the cutting process. In [18], the cutting studies showed that when cutting aluminum alloy, a tool force existed which did not act on the tool rake face but the tool nose. The results indicate that this force was independent of the depth of cut, the chip–tool contact length, and the chip thickness, under the cutting conditions investigated. Poletika [19] conducted several experiments on various metals, such as carbon, stainless steel, copper, and bronze, and found a unique relation between the chip thickness coefficient and the total contact length. Friedmann and Lenz [20] showed in their experiments that contact length and chip curl are affected by many parameters in the cutting system, and the influence of a single parameter is very difficult to isolate. Moreover, they concluded that their results support the hypothesis that contact length and chip curl variation are governed by the temperature field. Kato et al. [21], using the split tool method, reported that the contact length is twice the deformed chip thickness.

The natural contact length *l_C_* is divided into two areas where different contact conditions exist [3,5,19]: the plastic area and the elastic area. The first area is characterized by a plastic deformation process [3,22]. In the second area, an elastic frictional interaction takes place [3,6].

The natural contact length is the causal result of complex thermomechanical mechanisms and a variety of influencing factors. An investigation of the contact length can therefore only be carried out relatively. The methods and models developed in recent decades for determining the contact length of the tool rake face with the chip can be divided into three main areas:

- Experimental study;
- Analytical models;
- Numerical simulation.

The experimental measurements of contact length were generally performed by optical methods. Analyzing electron microscope photographs, Balaji and colleagues evaluated the contact length between the tool rake face and the chip in the sliding and sticking zones for grooved tools [10]. The dependence of the contact length on the tool’s thermal conductivity coefficient was established. Grzesik and Nieslony [23] measured the contact length by an imprint on the tool rake face, formed as a result of the cutting process. The measurements were performed using an optical image processing system for tools with different multilayer coatings. As a result, the dependence of the contact length on the cutting speed and the cutting temperature was obtained. Sutter’s study is devoted to measuring contact length during cutting using video recording with a high-speed camera [24]. The contact length was determined visually with a maximum error of 7%. The dependence of the contact length on the cutting depth (undeformed chip thickness) and the chip compression coefficient was compared with the calculated values of the contact length obtained by other authors.

Abukhshim et al., using an electron microscope, measured the contact length from the mark left by the chip on the tool rake face as a result of the high-speed cutting process [25]. The dependence of the contact length on the cutting speed at different values of tool wear was obtained. The investigations show that previous analytical models cannot adequately describe the contact length in HSM (high-speed cutting). Above 400 m/min, the natural contact length rises with the increasing cutting speed. Using a similar technique, Iqbal et al. studied the change in contact length as a function of cutting speed and undeformed chip thickness when cutting AISI 1045 steel and Ti6AlV4 titanium alloy [26]. The studies by Heisel et al. showed that reducing the contact length by artificially manipulating the rake face leads to a reduction in the cutting forces and a lowering of the chip compression ratio [27]. The reason for this is a shorter tribological chip–tool friction contact. This makes it possible to estimate the point at which the natural contact length is undercut. In [28], the authors used a Polyvar optical microscope to show that the machined material and the cutting speed also have a significant influence on the contact length. The study of the influence of contact length on the stability of the cutting process of structural steel and aluminum alloy is devoted by Ojolo and Awe [29]. Based on the obtained dependence of the contact length on the feed rate, the stability of the process was analyzed, and ways to increase it were indicated. The effect of ultrasonic vibration on the contact conditions of the tool rake face when cutting AISI 304 steel was studied by Amini and Kazemiyoun [30]. The contact length was determined by measuring the marks left by the chip on the tool rake face. Sadik und Lindström [31] considered the influence of artificially reduced contact lengths, with regard to the use of chip breaker grooves. The results showed that the technological contact length setting should be between the natural contact length and the plastic area of the entire contact zone. Ortiz-de-Zarate et al. [32] used a tool with a square groove in the rake face of the tool to adjust the contact length and to investigate the contact conditions in the cutting process.

The studies devoted to determining the contact length of the tool rake face with the chip by analytical methods occupy the major part of all research in this field. The methodological basis of these studies is the cutting theory; see, e.g., [1,2,5,15,22,33]. An overview of the numerous models for calculating the contact length of the tool rake face with chips and the expected trends in the development of the calculation models are presented in a study by Fatima and Mativenga [34].

By forcing the restrictions on contact length through a reduction in the length of the tool rake face, Oxley determined the shear angle and friction angle [35]. Analyzing the cutting process under these conditions, he predicted the dependence of the above characteristics on changes in the contact length. Friedman and Lenz have shown that the contact length is significantly influenced by the tool coating type with the constant basic cutting characteristics [20]. Chiffre and Wanheim proposed a mechanical model of chip formation that takes into account the stress distribution at the tool rake face [36]. This model can be the basis for the analytical calculation of the contact length. Poletika and Pushnykh considered two areas of contact length—plastic and elastic [37]. They proposed analytical expressions for determining the relative contact length for cutting processes with tools equipped with carbide inserts. Many authors use the slip-line method to determine the contact conditions between the tool rake face and the chip and, in particular, to determine the contact length; see, e.g., [15,38,39,40,41]. Fang used this method to study chip morphology during milling [42]. Aseev and Sidorenko have proposed a new mathematical model for calculating the total contact length and its plastic region [43]. The model is based on the assumption that chip shear occurs along the curve of the second order, which is confirmed experimentally. A model for calculating contact using a genetic algorithm was proposed by Zadshakoyan and Pourmostaghimi [44]. Evaluation of the tool–chip contact length for the orthogonal cutting process, using tools with multilayer coatings, is considered in a study by Balaji and Mohan [45]. The proposed model is based on a combination of analytical and empirical approaches and makes it possible to predict the contact length. Ozlu and his colleagues developed an analytical two-zone model of orthogonal cutting [46]. The model predicts the contact length, which coincides reasonably well with the experimental measurements. Ren et al. proposed a model for estimating contact length when cutting Ti6Al4V titanium alloy that takes into account the formation of segmented chips [47].

In the last few decades, studies have been carried out not only to simulate the main characteristics of the cutting process, such as the cutting force, cutting temperature, shear angle, chip compression coefficient, and chip morphology, but also to determine the contact length of the tool rake face with the chips through numerical models, mainly with FEM [48,49,50]. Semi-empirical models, such as the Johnson–Cook constitutive equation, have become the most popular for modelling different machining processes, due to their simplicity and capability in adequately describing flow curves in a wide variation range of the basic parameters. The Johnson–Cook constitutive equation represents, in a multiplicative form, the stress–strain relationship, which depends on five parameters [51]:

$$\sigma_{S}=\left(A+B\cdot \varepsilon^{n}\right)\cdot \left[1+C\cdot \ln\left(\frac{\dot{\varepsilon }}{{\dot{\varepsilon }}_{0}}\right)\right]\cdot \left[1\;-\;{\left(\frac{T-T_{0}}{T_{m}-T_{0}}\right)}^{m}\right]$$

where *σ_s_* is the yield point, *A* is the initial yield stress, *B* is the stress coefficient of strain hardening, *n* is the power coefficient of strain hardening, *C* is the strain rate coefficient, m is the power coefficient of thermal softening, *ε* is the strain, $\dot{\varepsilon }$ is the strain rate, ${\dot{\varepsilon }}_{0}$ is the reference value of the strain rate, *T* is the actual temperature, *T*_0_ is the reference or room temperature, and *T_m_* is the melting temperature of the material.

Bil and colleagues, implementing the finite element method, compared the numerical simulation results of the cutting process characteristics, including contact length, during orthogonal cutting for various commercial programs. The calculation results showed good agreement with the experimental data [52]. Analyzing various friction models in the contact of the tool rake face with chips by numerical simulation of the orthogonal cutting process, Iqbal et al. showed a significant dependence of the contact length on the chosen scheme of friction distribution in the tool rake face [53]. The Coulomb friction model and the sticking-sliding friction model were considered. Woon and colleagues analyzed the contact length in the micro-cutting process numerically and experimentally [54]. They showed good agreement between the values obtained by simulation and experimental studies using the optical method. Courbon et al. analyzed the thermal contact conditions between the tool, chip, and workpiece during the dry-cutting of AISI 1045 steel through numerical simulation and experimental studies using optical, electron microscopy and energy dispersive spectrometry methods [55]. The influence of the contact conductivity in the tool–chip pair on the contact length is shown. The authors recommend considering the average value of the contact length as significant fluctuations in the individual measurements of this value have been observed. Moreover, it was shown that the use of a high-pressure coolant resulted in a reduced natural contact length [56]. Kishawy and colleagues developed a thermoelastic–viscoelastic finite element model to simulate 304 L steel orthogonal cutting [57]. They used this model to investigate the influence of the cutting parameters and cutting tool geometry on the tool–chip contact conditions, particularly on the contact length. A significant influence of the feed rate on the contact length of the tool face with the chip was shown. In addition to the mere cutting investigation, numerical simulations can also provide input variables for analytical approaches [58].

The current state of knowledge shows the long development of the investigation of contact length in the cutting process. The large number of influencing parameters makes the simulative modeling of the contact length with the FEM very difficult. However, the simulation-based investigation of the thermomechanical and kinetic processes in metal cutting, as well as an a priori prediction of tool wear, can only be carried out with the correct contact length. This paper shows an approach for the inverse adaptation of the contact length in the simulation to the experimentally determined reference values in the cutting of AISI 1045.

## 3. Materials and Methods

### 3.1. Materials

**Figure 1:**
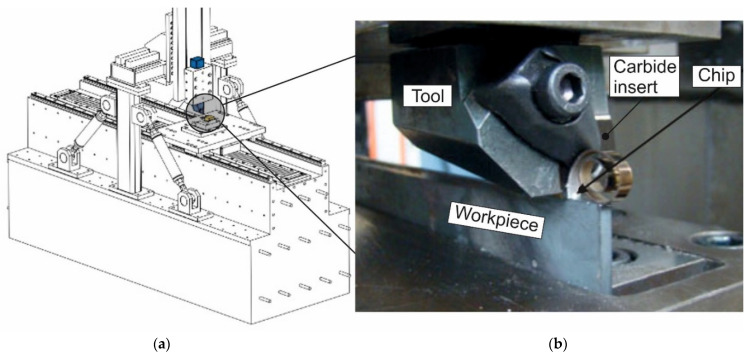

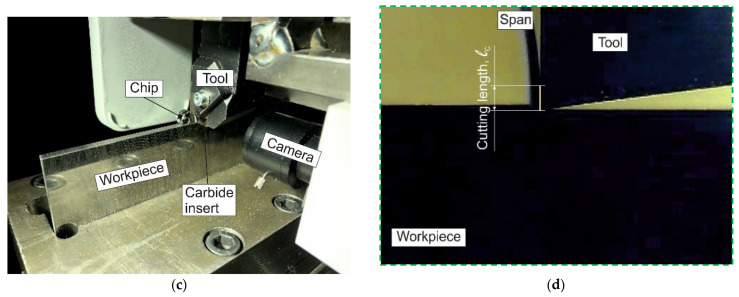
Experimental set-up for contact length measurement; CAD model (**a**); tool with the carbide insert (**b**); camera position in relation to the analyzed area (**c**); contact area (**d**).

Measurements of the contact length between the tool rake face and the chip were performed on a special experimental stand that realizes the process of orthogonal and oblique cutting—Figure 1 [59,60].

The CAD model of the experimental stand is shown in Figure 1a. The bed of the test stand is manufactured from solid polymer concrete. On the bed, there are slideways for moving the table with the workpiece. To measure the cutting forces on the workpiece side, it was clamped in a three-component dynamometer type 9121 from Kistler, which is located on the table. The linear motor provides the reciprocating motion of the table. The table movement speed with the workpiece is continuously adjustable from 0 to 200 m/min. This covers the conventional cutting speed range for most metals and alloys. The drive system consists of a linear motor, a servo control unit, including power electronics, and a computer as the control system.

The tool holder is fixed to the gantry structure connected to the bed. To increase the rigidity, the gantry with the tool is additionally connected to the bed using tension rods, the prestressing of which can be adjusted. This adjustment is made when it is necessary to change the stiffness of the experimental stand. The required cutting depth is ensured by adjusting the movement of the ram and the tool holder attached to it to an accuracy of 0.01 mm. SNMG-SM-1105 (ISO SNMG 15 06 12-SM 1105), Sandvik Coromant, Sandviken, Sweden interchangeable carbide inserts were used as a cutting element. The cutting wedge geometry required for cutting was provided by positioning the carbide insert in the tool body at a specified rake angle and grinding the tool’s clearance face. The tool’s clearance angle was 8°, and the radius of the cutting-edge rounding was 20 µm in all tests. A view of the tool with the carbide insert while cutting the workpiece is shown in Figure 1b.

Orthogonal cutting was performed on workpieces from AISI 1045 steel with a thickness of 3 mm, a length of 170 mm, and a height of 60 mm. To achieve the necessary geometric accuracy, the edges of the workpiece were pre-ground and polished [61,62]. To remove residual stresses, the workpiece was subjected to thermal annealing. After annealing, the hardness of the workpiece was HB 1700–1750 MPa. The mechanical and thermal properties of this material are listed in Table 1.

The experimental studies were performed during dry orthogonal cutting. During the experiments, three values of the tool rake angle were used: −10°, 0°, and 10°. The cutting process of the AISI 1045 steel was analyzed for varying cutting speeds and undeformed chip thicknesses (depth of cut). Varying the cutting speed from 30 m/min to 180 m/min and the depth of cut from 0.1 to 0.25 mm was implemented so that the Péclet similarity criterion (Péclet number) [7,27] changed from ca. 5 to ca. 100.

### 3.2. Methods

The camera position in relation to the analyzed contact area of the tool rake face with the chip is presented in Figure 1c. An example of a camera image from the contact area used for contact length analysis is shown in Figure 1d. The camera’s resolution provides multiple separate images during a tool pass. The uncertainty in the contact length measurement arises mainly from the fluctuations of the chip during the cutting process. Because of this, the coordinates of the last point of chip contact with the tool face are constantly changing. This uncertainty can be eliminated by repeating the measurements multiple times. Repeating separate cuts multiple times (at least 5 times), together with the analysis of separate images of one cut, provided a measurement error in the contact length of the tool rake with the chip within 6–11%, depending on the cutting speed.

## 4. Results of Contact Length Experimental Measurement

**Figure 2:**
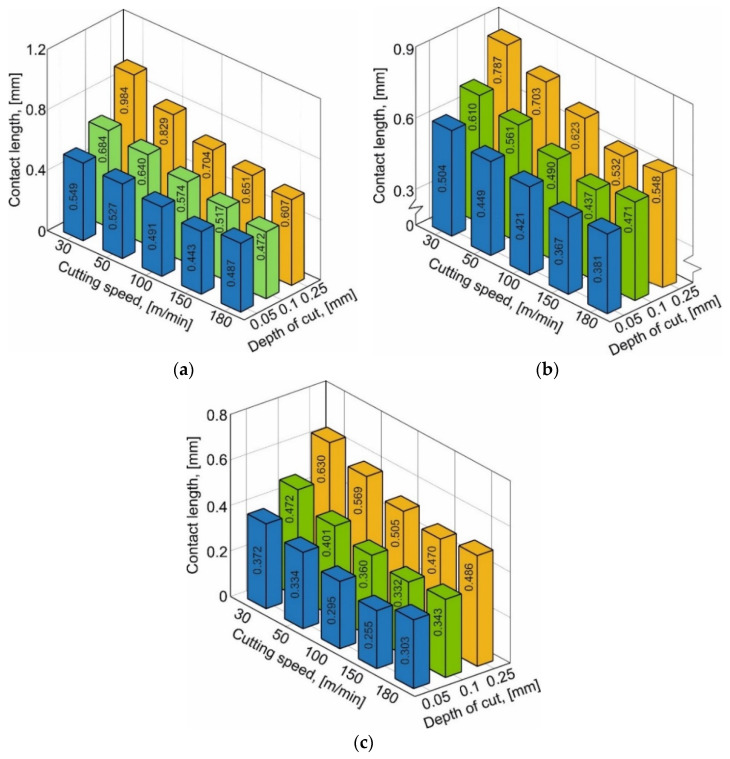
Contact length depends on cutting speed and depth of cut. (**a**) γ = −10°, (**b**) γ = 0°, (**c**) γ = 10°.

The measurement results of the contact length between the tool rake and chip that depend on the cutting speed and depth of cut (undeformed chip thickness) and the cutting wedge rake angle of the tool are shown in Figure 2. The contact length changes show a similar character of dependence on the cutting speed and depth of cut for all three studied tool rake angles. Thus, the contact length monotonically decreases with the increasing of the cutting speed. With the increase in cutting depth, the contact length increases monotonically for all three values of the tool rake angle (see Figure 2a–c). The exception to this monotonic variation is the contact length values at a cutting speed of 180 m/min. This is due to dynamic phenomena when cutting with such high cutting speeds. At significant values of the cutting speed, the inertial properties of the chip are observed. As a result of inertial forces, the chip is pressed against the tool rake face, thereby increasing the contact length.

However, this results in relatively small deformation values of the machined material, which are achieved during cutting using tools with rake angles of 0° and 10°. With large values of material deformation provided during cutting with a tool rake angle of −10° or higher, the chips formed are stronger and more stable. As a result, the stability of the chip prevails over the inertial forces. However, with a relatively low depth of cut and, consequently, a small chip thickness, the inertial forces prevail over the strength and stability of the chip formed during cutting with a tool rake angle of −10°. This is indicated by the increased contact length at a cutting speed of 180 m/min and a cutting depth of 0.05 mm (see Figure 2a).

In addition to the dynamic behavior of the technological system, other factors have a significant influence on the contact length deviation, such as, in particular, the instability of the tool position relative to the machined surface caused by the spring back [65], and many others. This causes some uncertainty in the measurement of the contact length by optical methods. This uncertainty is eliminated by repeating measurements at each point of change in the cutting conditions and tool geometry.

To analyze the combined effect of the cutting parameters and the tool cutting wedge geometry on the contact length, it is necessary to present the studied results in the form of a generalized dependence.

The experimental results of the orthogonal cuts show that all three investigated variables have a decisive influence on the contact length between the chip and the tool. Due to these numerous influencing factors, it is reasonable to describe the relationship between the influencing variables of cutting speed, cutting depth (undeformed chip thickness), and tool rake angle and the target variable of the contact length with an empirical equation. A range of different approaches can be used for this purpose. Particularly suitable in this regard are those function types that calculate the target value as the product of several potency functions with the respective parameter in the base and an exponent.

Each potency function has a weighting factor which brings the effect of the individual influence factors into equilibrium. This type of function is also used to describe the contact length as a function of the influence quantities investigated in this work:

(1) $$l_{C}=D_{1}\cdot V_{c}^{x}\cdot D_{2}\cdot a^{y}\cdot D_{3}\cdot {\left(360{}^{\circ}+\gamma \right)}^{z}=(D_{1}\cdot D_{2}\cdot D_{3})\cdot V_{c}^{x}\cdot a^{y}\cdot {\left(360+\gamma \right)}^{z}=\;D\cdot V_{c}^{x}\cdot a^{y}\cdot {\left(360+\gamma \right)}^{z}$$

where $D=D_{1}\cdot D_{2}\cdot D_{3}$.

Compared to the additive function types, the multiplicative function character takes into account the mutual interactions between the individual parameters. Furthermore, the potency function has a comparatively high physical realism at the lower interval limit. Thus, if there is no relative movement between the active partners, no cutting process takes place, which is why no chip is produced. The same applies if the depth of cut (undeformed chip thickness) assumes a value of zero. The tool would slide over the workpiece without forming a chip. When considering the tool geometry, it makes sense to limit the rake angle. Theoretically, a cutting process can take place if the rake angle varies in the range between −90° and +90°. Therefore, the following limitation of the influencing variables results from the discussion carried out:

(2) $$\left\{ \begin{array}{l} V_{C}\geq 0\;m/\min\\ a\geq 0\;\mathrm{mm}\\ -90{}^{\circ}<\gamma <90{}^{\circ} \end{array}\right.$$

To find the function parameters, the formula is linearized using the logarithm operator, and the parameters are determined using suitable interpolation points. To cover a wide factor space, the parameter limits defined above can be used. However, from a practical point of view, it can be stated that the choice of interpolation points at the parameter margin is not expedient as the contact length is also zero at the cutting speed *V_c_* = 0 m/min or the cutting depth *a* = 0 mm. The rake angles at the parameter margin would also not lead to any meaningful results, which is why a further, reasonable restriction of the parameter space must be made. This restriction may be based on common cutting data and tool geometries and can be derived, e.g., from catalogs. As result of catalog research and considering the experimentally realizable values, the factor space is narrowed down as follows:

(3) $$\left\{ \begin{array}{l} 30\;m/\min\leq V_{C}\leq 150\;m/\min\\ 0.05\;\mathrm{mm}\leq a\leq 0.25\;\mathrm{mm}\\ -10{}^{\circ}<\;\gamma <10{}^{\circ} \end{array}\right.$$

The factors *x*, *y*, *z*, and *D* are determined by calculating the zeros of the linearized function of the partial derivatives. The outer parameter values serve as supporting points. The calculated function parameters are shown in Table 2. The derived approximation equation serves to quantitatively describe the contact length as a function of the defined parameters. However, a comparative analysis of the sensitivity of the individual parameters based on the exponents is not possible as the scales describe different physical quantities. In the formula, this difference is corrected by the multiplier *D*. The multiplier *D* is the product of the multipliers *D*_1_, *D*_2_, *D*_3_, etc.

An estimation of the tendency of the parameters to influence the contact length can be made based on the analysis of the exponents. As the amount of all the exponents is below the value of one, the contact length changes disproportionally with the increase in the parameters. Due to the minus sign of the factors *x* and *z*, increasing the cutting speed and the rake angle results in a reduction of the contact length. If the cutting depth is increased, the contact length rises.

## 5. Results and Discussion of Contact Length Determination Using Numerical Simulation

Determining the contact length experimentally (see Section 4) causes significant difficulties in realizing such measurements. In addition to the need for extensive studies, the direct measurement of the contact length is also limited by the considerable uncertainty in the determination of the end coordinates of the chip contact with the tool rake face, i.e., the coordinates of the chip breakaway from the rake face. The use of numerical methods for modeling the cutting process, the finite-element methods in particular, provides a significant reduction in the labor intensity of determining the contact length. To simulate the contact between the tool rake face and the chip, a FEM model of cutting in a two-dimensional formulation was developed.

The FEM model of cutting was developed to simulate cutting processes based on the updated implicit Lagrangian formulation method. It was assumed that the material of the workpiece was isotropic of the plastic type. The material of the tool was rigid. The material model of the steel AISI 1045 was based on the constitutive Johnson–Cook equation with the basic model parameters listed in Table 3 [63,66].

**Figure 3:**
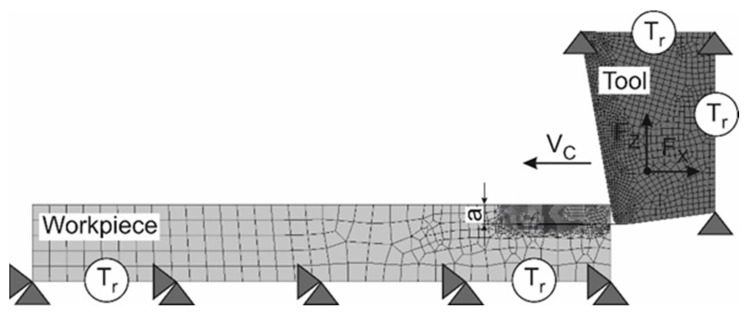
Initial geometry, boundary conditions, and mesh of the FE cutting model.

The contact between the tool and the chip, as well as between the tool and the workpiece, was modeled with a hybrid friction model. The friction model was composed of a combination of the Coulomb model and a shear friction model [67]. The meshed initial geometrical model with boundary conditions for a tool with a rake angle of −10° is shown in Figure 3. For the tools with other rake angles, the FE model is similar.

The boundary conditions were determined by fixing the workpiece and the tool as well as by the input of the thermal conditions at the boundaries of the respective objects. The bottom of the workpiece was rigidly fixed in the X- and Y-directions. The thermal initial conditions at room temperature *T_r_* were given at the bottom and the lefthand side of the workpiece as well as at the righthand side and the top of the tool. The working motion of the tool at a cutting speed *V_C_* for guaranteeing the cutting process was given by the absolute motion in the negative X-direction. The depth of cut (undeformed chip thickness) was determined by the value *a*.

Multiple studies have shown that the material model and friction model parameters have a significant influence on the contact length of the tool rake face with the chip; see, e.g., [1,12,25]. The parameters of the selected hybrid friction model were determined based on the measured kinetic characteristics of the cutting process according to the methodology [68]. Previously, the prevailing effect of the constitutive equation parameters on the contact length of the tool rake face with the chip was found to be predominant [59,69,70].

**Figure 4:**
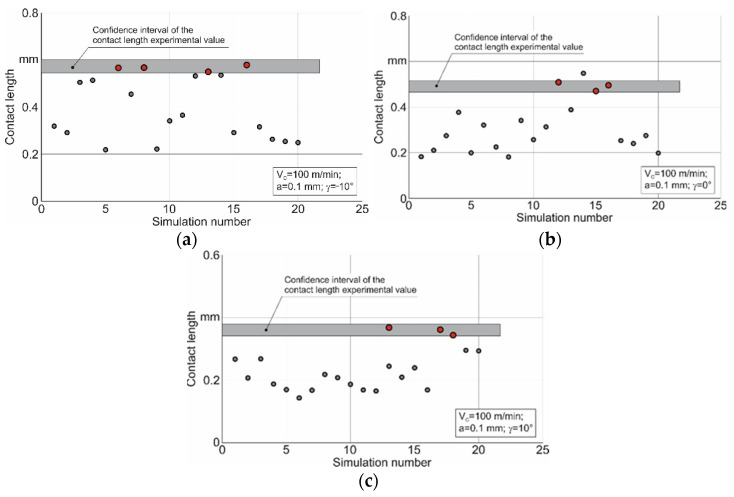
First iteration results of determining the constitutive equation parameters. (**a**) γ = −10°, (**b**) γ = 0°, (**c**) γ = 10°.

The parameters of the constitutive equation were determined by repeated DOE (design of experiments) iterations; see, e.g., [61,66]. The first iteration results of determining the Johnson–Cook constitutive equation parameters for the studied three values of the tool rake angles are shown in Figure 4.

In this iteration, the parameter limits of the constitutive equation are set over a wide range of possible values. Within the confidence interval of the contact length, the experimental values for the tool rake angle, and the cutting modes indicated in the diagram, a rather small number of parameter sets of the constitutive equation fall into the confidence interval. These parameter sets are marked with red circles on the diagrams. Apparently, the small number of the parameter sets that are within the confidence interval of the experimental values for contact length indicates that the interval of the constitutive equation parameters that provide contact length values corresponding to its experimental value is also rather limited.

**Figure 5:**
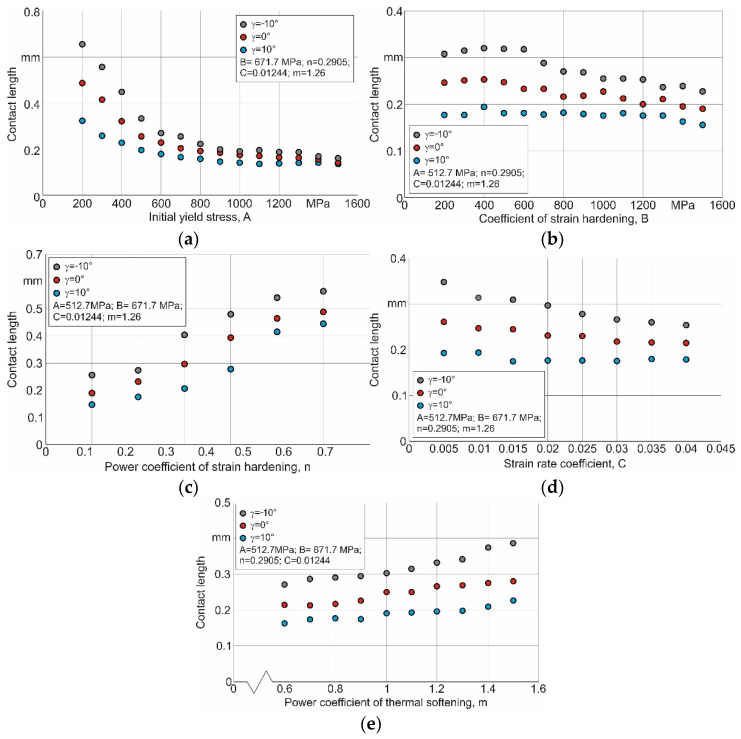
Effect of separate parameters on the contact length. (**a**) initial yield stress *A*, (**b**) strain-hardening coefficient *B*, (**c**) power factor of stress hardening *n*, (**d**) strain rate coefficient *C*, (**e**) power factor of thermal softening *m*.

To refine such suitable intervals for varying the constitutive equation parameters, it is necessary to evaluate the effect nature of the individual parameters on the contact length value. Figure 5 shows this effect.

When the individual parameters of the constitutive equation change within the wide ranges of their values, the initial yield stress *A*, the power factor of stress hardening *n*, and the power factor of thermal softening *m* have a clear influence on the contact length. With the increasing *A* value, the contact length monotonically decreases, approaching a minimum constant value (see Figure 5a). In this case, the *A* values that provide the contact lengths corresponding to their experimental values are in the range of 300 MPa to 600 MPa. As the value of the coefficient *n* increases, the contact length also increases significantly (see Figure 5c). The *n* values, which ensure that the contact lengths correspond to their experimental values, are in the range of 0.35 to 0.6. The decrease in the softening of the machined material, which increases as the coefficient *m* increases, leads to a certain increase in the contact length (see Figure 5e). The values of the coefficient *m*, which provide contact lengths corresponding to their experimental values, are in the zone of the large values of this coefficient, starting from the value of 1.2. The influence of the strain-hardening coefficient *B* and strain rate coefficient *C* on the contact length for the studied values of the tool rake angles is uncertain (see Figure 5b,d).

The effect nature of the separate parameters of the Johnson–Cook constitutive equation on the contact length and the limits of their suitable values will certainly change with the simultaneous changes in all the equation parameters, which is the case when carrying out DOE. However, analysis of the influence of the separate parameters on the contact length contributes to determining the approximate variation limits for the separate parameters needed to perform the second iteration of the DOE.

**Figure 6:**
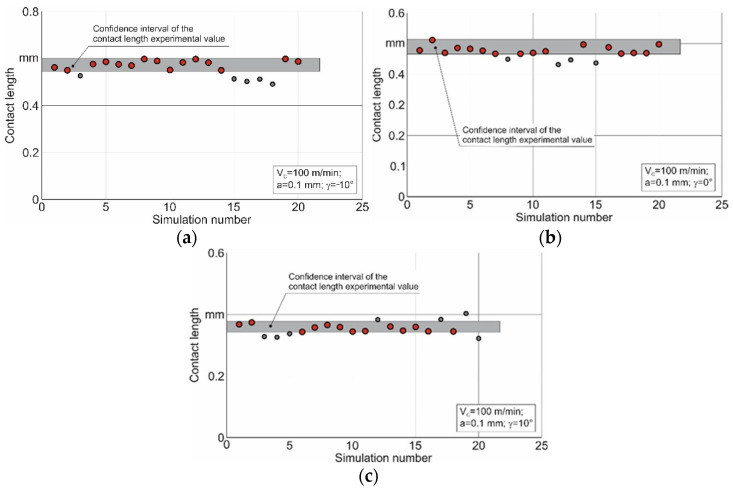
Second iteration results of determining the constitutive equation parameters. (**a**) γ = −10°, (**b**) γ = 0°, (**c**) γ = 10°.

The limits of the parameters determined by such an analysis are presented in Table 4. Using the values of the upper and lower limits of variation for the constitutive equation parameters given in Table 4, the second iteration of the DOE was carried out. The results of this iteration for the studied tool rake angles are shown in Figure 6.

A substantially larger number of different sets of the constitutive equation parameters came within the confidence interval of the contact length experimental values as a result of the second iteration. These parameter sets are indicated on the diagrams with red circles.

Thus, for each value of the tool rake angle considered in this study, there is some set of constitutive equation parameters that provides a numerically simulated contact length approximately corresponding to its experimental value. The generalized values of the constitutive equation parameters satisfying all the studied values of the tool rake angles may be determined by finding the intersection of the pluralities of the parameters defined as a result of the second iteration (see Figure 6). Naturally, these generalized values can be defined only if the plurality intersection is not empty. In the case of an empty plurality, the algorithm for determining the generalized parameters of the constitutive equation should provide for the redefining of the parameter limits for subsequent iterations and possibly the increasing of the number of simulations. A similar methodology for finding the multiple plurality intersections of the constitutive equation parameters can also be applied to any cutting characteristic or to a combination of several cutting characteristics obtained by the convolution product of individual characteristics.

The software algorithm developed based on this methodology provides a finding of the required plurality intersection of the constitutive equation parameter set. The developed algorithm implements redefinition and refinement of the parameter limits for successive simulation iterations in the detection of an empty plurality intersection of parameter sets. One of the most acceptable variants of the set of the constitutive equation parameters, determined using the described algorithm, is listed in Table 5.

**Figure 7:**
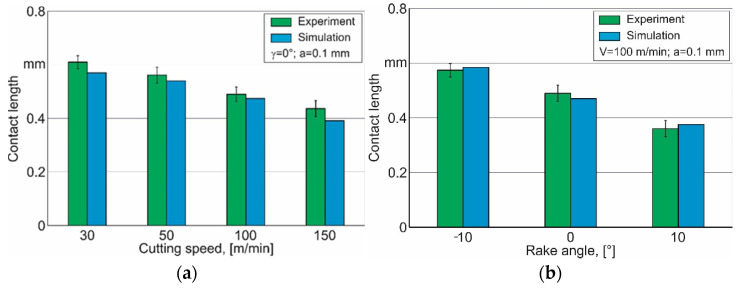

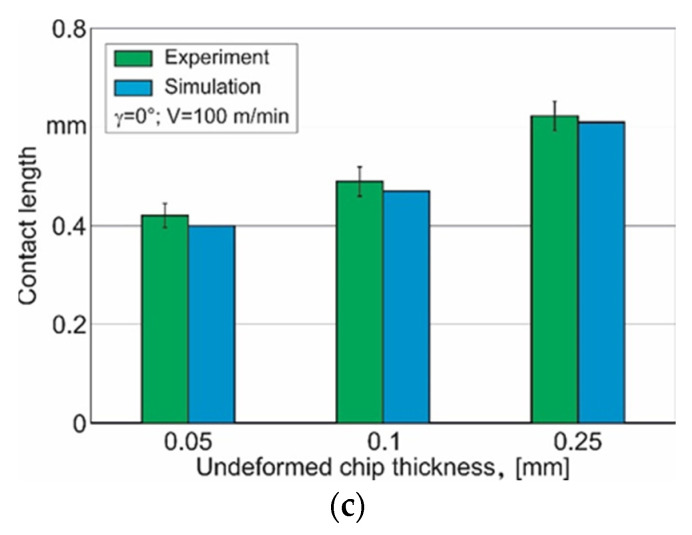
Dependence of simulated contact length on cutting modes and tool rake angle. (**a**) γ = −10°, (**b**) γ = 0°, (**c**) γ = 10°.

Using the parameter values presented in Table 5, the contact length during cutting was simulated at different cutting speeds and depths of cut, as well as the tool rake angles. Exemplarily, Figure 7 presents the dependency of the contact length on these values.

The largest deviation of the simulated contact length from its experimental value at different cutting speeds was 12% and is observed at a cutting speed of 150 m/min—Figure 7a. For the other examined values of cutting speed, the deviation of the simulated contact length from its experimental value does not exceed 9%. Therefore, a significant increase in the cutting speed leads to an increase in the deviation of the simulated contact length values from its experimental value. The further increase in the cutting speed and transition to high-speed cutting is expected to lead to a significant deviation between the simulated and the experimentally determined contact length values. This is most likely due to the fact that the stress–strain state of the machined material in all the cutting zones and, in particular, in the secondary cutting zone is predominantly influenced by the cutting temperature. Increasing the cutting temperature at high cutting speeds leads to higher degrees of machined material softening and, thus, reduces the simulated contact length between the tool rake face and the chip. The difference in the simulated and experimental values of the contact length at different tool rake angles is approximately the same and does not exceed 4%—Figure 7b. The largest difference between the simulated contact length and its experimental value when changing the cutting depth is 4.3% and is observed at a cutting depth of 0.05 mm—Figure 7c.

Thus, the generalized set of the constitutive equation parameters, determined using the developed software algorithm, provides an insignificant deviation of the simulated contact length values from their experimental values when changing the cutting modes and tool geometry within a relatively wide range.

## 6. Conclusions

A methodology for determining the contact length of the tool rake face with the chip, using a numerical simulation of the orthogonal cutting process for metals that form flow chips, is proposed. This methodology is based on determining the generalized parameters of the constitutive equation as the contact length is predominantly influenced by the constitutive equation parameters describing the model of the material to be machined. The use of this methodology provides a determination of the contact length for a wide range of cutting modes and tool geometries, particularly the tool rake angle.

For the determination of the generalized parameters of the constitutive equation, a software-implemented algorithm has been developed. The algorithm is based on finding the plurality intersection of these parameter sets. The sets of constitutive equation parameters are determined by DOE (design of experiments) and refined by subsequent multiple iterations.

The validity of the constitutive equation parameters was evaluated by comparing the simulated values of the contact length with its experimental values. The experimental value of the contact length with varying cutting speed, depth of cut, and tool rake angle was performed by an optical method. The contact length obtained by simulation of the cutting process, using the generalized parameters of the constitutive equation as a material model, differs from its experimental value by an amount not exceeding 12%.

The developed methodology for determining the total tool–chip contact length is limited to the class of metals that provide the formation of flow chip and machining methods based on orthogonal free cutting. For the future, the plan is to extend the developed methodology to the processes of oblique free cutting and oblique non-free cutting.

## Figures and Tables

**Table 1:** Mechanical and thermal properties of the steel AISI 1045 [63,64].

Strength (MPa)		Elastic Modulus (GPa)	Elongation (%)	Hardness (HB)	Poisson’s Ratio	Specific Heat (J/kg·K)	Thermal Expansion (µm/m·°C)	Thermal Conductivity (W/m·K)
Tensile	Yield							
690	620	206	12	180	0.29	486	14	49.8

**Table 2:** Approximation equation parameters.

x	Y	z	D
−0.20452	0.30876	−0.0035232	8.193

**Table 3:** Initial parameters of the Johnson–Cook constitutive equation.

Constitutive Parameters				
*A* [MPa]	*B* [MPa]	*n*	*C*	*m*
512.3	671.7	0.2905	0.01244	1.26

**Table 4:** Variation limits for the constitutive equation parameters.

Constitutive Parameters									
*A* [MPa]		*B* [MPa]		*n*		*C*		*m*	
Upper limit	Lower limit	Upper limit	Lower limit	Upper limit	Lower limit	Upper limit	Lower limit	Upper limit	Lower limit
900	400	1100	700	0.49	0.42	0.015	0.008	1.2	0.8

**Table 5:** Generalized values of the constitutive equation parameters.

Constitutive Parameters				
*A* [MPa]	*B* [MPa]	*n*	*C*	*m*
720	895	0.475	0.012	0.98

## Data Availability

Not applicable.
